# Non‐invasive vagus nerve stimulation to reduce ileus after colorectal surgery: randomized feasibility trial and efficacy assessment (IDEAL Stage 2B)

**DOI:** 10.1111/codi.17194

**Published:** 2024-10-12

**Authors:** Stephen J. Chapman, Mikolaj Kowal, Jack A. Helliwell, Sonia Lockwood, Maureen Naylor, Julie Croft, Katherine Farley, Deborah D. Stocken, David G. Jayne

**Affiliations:** ^1^ Leeds Institute of Medical Research University of Leeds Leeds UK; ^2^ Bradford Teaching Hospitals NHS Foundation Trust Bradford UK; ^3^ West Yorkshire Ileostomy Association UK; ^4^ Clinical Trials Research Unit, Leeds Institute of Clinical Trials Research University of Leeds Leeds UK; ^5^ Leeds Institute of Health Sciences University of Leeds Leeds UK

**Keywords:** clinical trials, gastrointestinal surgery, intestinal motility, nerve–gut interactions

## Abstract

**Aim:**

Ileus is characterized by a period of intestinal dysmotility after surgery, leading to vomiting and constipation. In preclinical models, vagus nerve stimulation reduces intestinal inflammation and prevents smooth muscle dysfunction, accelerating the return of gut function. This study explored the feasibility of a definitive trial of non‐invasive vagus nerve stimulation (nVNS) along with an early assessment of efficacy.

**Method:**

A multicentre, randomized feasibility trial (IDEAL Stage 2B) of self‐administered nVNS was performed. Patients undergoing colorectal surgery were randomized to nVNS or sham before and after surgery. Feasibility outcomes comprised assessments of recruitment, compliance, blinding and attrition. Clinical outcomes were measures of intestinal function and adverse events. All participants were followed up for 30 days. Interviews with patients and health professionals explored barriers to feasibility and perspectives around implementation.

**Results:**

In all, 125 patients were approached about the study and 97 (77.6%) took part. Across all randomized groups, the median compliance to treatment was 19 out of 20 stimulations (interquartile range 17–20). The incidence of adverse events was similar across groups. In this unpowered feasibility study, the time taken for the return of gut function (such as first passage of stool) was similar between nVNS and sham treatments. According to interviews, patients were highly motivated to use the device because it provided them with an opportunity to engage actively in their care. Health professionals were highly driven to tackle the problem of ileus.

**Conclusion:**

Powered assessments of clinical efficacy are required to confirm or refute the promise of nVNS, as already demonstrated in preclinical models. This feasibility study concludes that a definitive randomized assessment of the clinical benefits of nVNS is desired and feasible.

**What does this paper add to the literature?**

Postoperative ileus is a common complication after colorectal surgery. Non‐invasive vagus nerve stimulation is a new candidate treatment. This study reports essential feasibility data, enabling the current work around this topic to progress from its focus on preclinical assessments of efficacy to randomized clinical assessments of efficacy and clinical effectiveness.

## INTRODUCTION

Postoperative ileus is a temporary cessation of coordinated intestinal motility after surgery. It is characterized clinically as a disruption of normal bowel function, leading to painful abdominal distension, vomiting and constipation which may persist for up to 10 days [1]. Owing to its profound impact on patients and healthcare systems, the prevention and management of ileus is considered as being amongst the highest research priorities in the field of colorectal surgery [2]. Whilst numerous clinical interventions to reduce ileus after surgery have been explored in the past, few have shown sufficient clinical promise to be implemented in clinical practice [3].

The development of ileus comprises two distinct phases: an early neurogenic phase and a later inflammatory phase. During the early phase, stimuli elicited by the initial peritoneal incision trigger a sympatho‐sympathetic inhibitory reflex in the spinal cord, transiently abolishing intestinal motility. Handling of the gut then activates mast cells, leading to increased mucosal permeability, bacterial translocation and activation of intestinal macrophages. In the later phase, activated macrophages release pro‐inflammatory mediators, attracting nitric‐oxide‐releasing leucocytes and leading to impaired contractility of intestinal smooth muscle [4]. Preclinical studies have shown that vagus nerve stimulation activates a cholinergic anti‐inflammatory pathway which suppresses intestinal inflammation, prevents smooth muscle dysfunction and in turn reduces ileus. This is mediated by nicotinic acetylcholine receptors residing on intestinal macrophages which activate the Jak2‐STAT3 signalling pathway [5, 6, 7, 8].

A small number of studies have explored vagus nerve stimulation in patients undergoing surgery. Some groups have assessed the role of invasive stimulation, demonstrating its anti‐inflammatory properties and showing that the technique can be performed without major adverse events [9]. Others have explored non‐invasive approaches, such as stimulation of the auricular vagus nerve, confirming that a non‐invasive approach successfully stimulates efferent vagal fibres [10, 11]. Building on this, we previously demonstrated proof‐of‐concept for non‐invasive stimulation of the cervical vagus nerve (nVNS) using a self‐administered device [12].

This study explores the feasibility of self‐administered nVNS across two clinical centres to optimize key study methods for a definitive trial and to explore preliminary efficacy for reducing postoperative ileus. This will build on the growing body of preclinical and early clinical work, providing an argument for or against a definitive, powered, randomized assessment of nVNS.

## METHODS

### Study summary

A multicentre, randomized, feasibility assessment of nVNS to reduce ileus after colorectal surgery was performed (ISRCTN62033341). Ethics approval was confirmed by the Tyne and Wear South NHS Research Ethics Committee (19/NE/0217). The study enrolled participants between 1 January 2020 and 31 December 2022. Interviews with patients and health professionals were undertaken to explore attitudes and perspectives towards a future trial and to implementing nVNS in practice. The study is reported according to the CONSORT 2010 Extension for Pilot and Feasibility Trials and the protocol was published previously [13, 14].

### Study setting and participants

The study was performed at two large hospitals in the UK (St James's University Hospital, Leeds, and Bradford Royal Infirmary, Bradford). At both sites, participants were treated within fast‐track recovery programmes alongside usual anaesthetic protocols [15]. Adult patients undergoing minimally invasive colorectal resection with no anticipated plan for a diverting stoma were eligible. Patients with severe cardiac or cerebrovascular disease, an implanted stimulator device, recurrent episodes of syncope, vagotomy, inflammatory bowel disease or an existing stoma were excluded. The full eligibility criteria are reported in Supplement S1.

### Interventions

**FIGURE 1:**
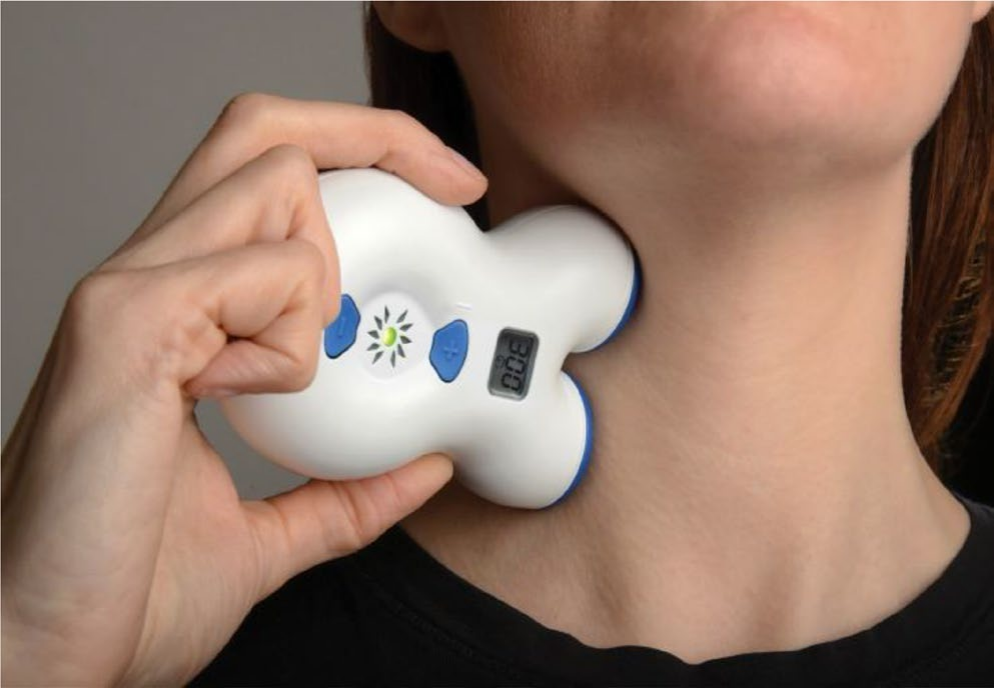
Positioning of non‐invasive vagus nerve stimulator (nVNS) device. Image provided courtesy of electroCore LLC.

The gammaCore device (electroCore Inc., NJ, USA) is a non‐invasive, self‐administered, electrical device used for cervical vagus nerve stimulation (Figure 1). It produces a low‐voltage stimulus comprising a 5‐kHz sine wave burst lasting for 1 ms (five waves of 200 μs) repeated every 40 ms (25 Hz). This generates a maximum voltage of 24 V and peak current of 60 mA which users can manually adjust. The sham variant produces a stepped‐square pulse repeated at 0.1 Hz with a maximum voltage of 6 V and maximum current of 2.7 mA. It is identical in appearance, weight, user interface and packaging. All participants took part in a mandatory training session (Supplement S1), after which they self‐administered the device twice daily for five consecutive days before and after surgery (total 10 days, irrespective of the time of first intestinal function). Each administration comprised a 2‐min stimulation cycle performed sequentially on each side of the neck. Participants were instructed to adjust the stimulation amplitude to the highest tolerated level.

### Feasibility trial

#### Study design

A multicentre, participant‐blinded, sham‐controlled, randomized, feasibility trial was performed with participants randomized across four parallel treatment groups (1:1:1:1):

- Group 1: Preoperative stimulation and postoperative stimulation (+, +)
- Group 2: Preoperative stimulation and postoperative sham (+, –)
- Group 3: Preoperative sham and postoperative stimulation (−, +)
- Group 4: Preoperative sham and postoperative sham (−, –)

Randomization was performed centrally using a 24‐h randomization system developed by the Leeds Clinical Trials Research Unit. This used minimization with two stratification variables, comprising type of surgery (right‐ or left‐sided resection) and hospital site. Participants were blinded to the allocation using an identical sham device.

#### Study outcomes and measures

Feasibility outcomes included participant recruitment, blinding performance according to the Bang blinding index, treatment compliance using self‐reported diaries, and rate of attrition [16]. These were assessed using a series of a priori progression criteria (Table 1). Clinical outcomes comprised time to first flatus, stool, oral intake and intestinal recovery (GI‐2) as well as the need for nasogastric intubation and length of hospital stay [17]. Adverse events and complications were measured at 30 days and reported using the Clavien–Dindo classification [18].

**TABLE 1:** Feasibility progression criteria.

Progression criteria	Stop	Modify	Go
Proportion of eligible patients identified from screening logs	<10%	10%–20%	>20%
Number of eligible patients randomized (site: SJUH)	≤2 per month	3–4 per month	≥5 per month
Number of eligible patients randomized (site: BRI)	<1 per month	1–2 per month	≥3 per month
Adequacy of participant blinding (according to the Bang blinding index)	Index < −0.5	Index −0.2 to −0.5	Index 0 to −0.19
	Or index >0.5	Or index 0.2–0.5	Or index 0–0.19
Average participant‐reported compliance to the study treatment schedule	<10/20 stimulations across 10 days	10–15/20 stimulations across 10 days	≥16/20 stimulations across 10 days
Rate of randomized patients lost to follow‐up	≥40%	15%–39%	<15%
Incidence of complications or serious complications	>20% increase in complications	5%–20% increase in complications	<5% increase in complications

Abbreviations: BRI, Bradford Royal Infirmary, Bradford, UK; SJUH, St James's University Hospital, Leeds, UK.

#### Sample size and data analysis

The literature recommends a sample size of at least 70 participants in external pilot trials [19]. The study was not powered to detect any differences in clinical efficacy outcomes but rather was designed to explore possible signals of benefit. All analyses were performed using the intention‐to‐treat population. Feasibility and clinical data are presented descriptively as rates (categorical) and means (continuous) as appropriate. Blinding performance was evaluated using the Bang blinding index, expressed as a value between −1 and 1 (0 implying random guessing).

#### Public involvement

A public advisory group chaired by an experienced patient representative guided the design and conduct of the study, as well as the interpretation of study results. The group met at predefined way‐points during the course of the feasibility study, contributing to study training processes as well as iterative refinements to the recruitment strategy.

### Participant interviews

An embedded qualitative study comprising semi‐structured interviews was undertaken. All patients approached for participation in the feasibility trial (irrespective of their decision to enrol) as well as surgeons and specialist nurses practising in any UK hospital were eligible. Participants were sampled purposively with consideration to age and sex until the data were saturated [20]. All participants took part in one interview. A thematic framework analysis was undertaken with consideration to the theoretical framework of acceptability [21, 22]. A single investigator (SC) reviewed all transcripts and a small number were independently reviewed by an independent researcher (KF) as a means of validation. An initial coding framework was generated from early transcripts and field notes, which was iteratively adapted as new data emerged. The final framework was used to construct themes through a process of mapping and cross‐comparison to explore between‐theme relationships. An abbreviated summary of key data is reported along with representative verbatim quotations.

## RESULTS

### Participant characteristics

**FIGURE 2:**
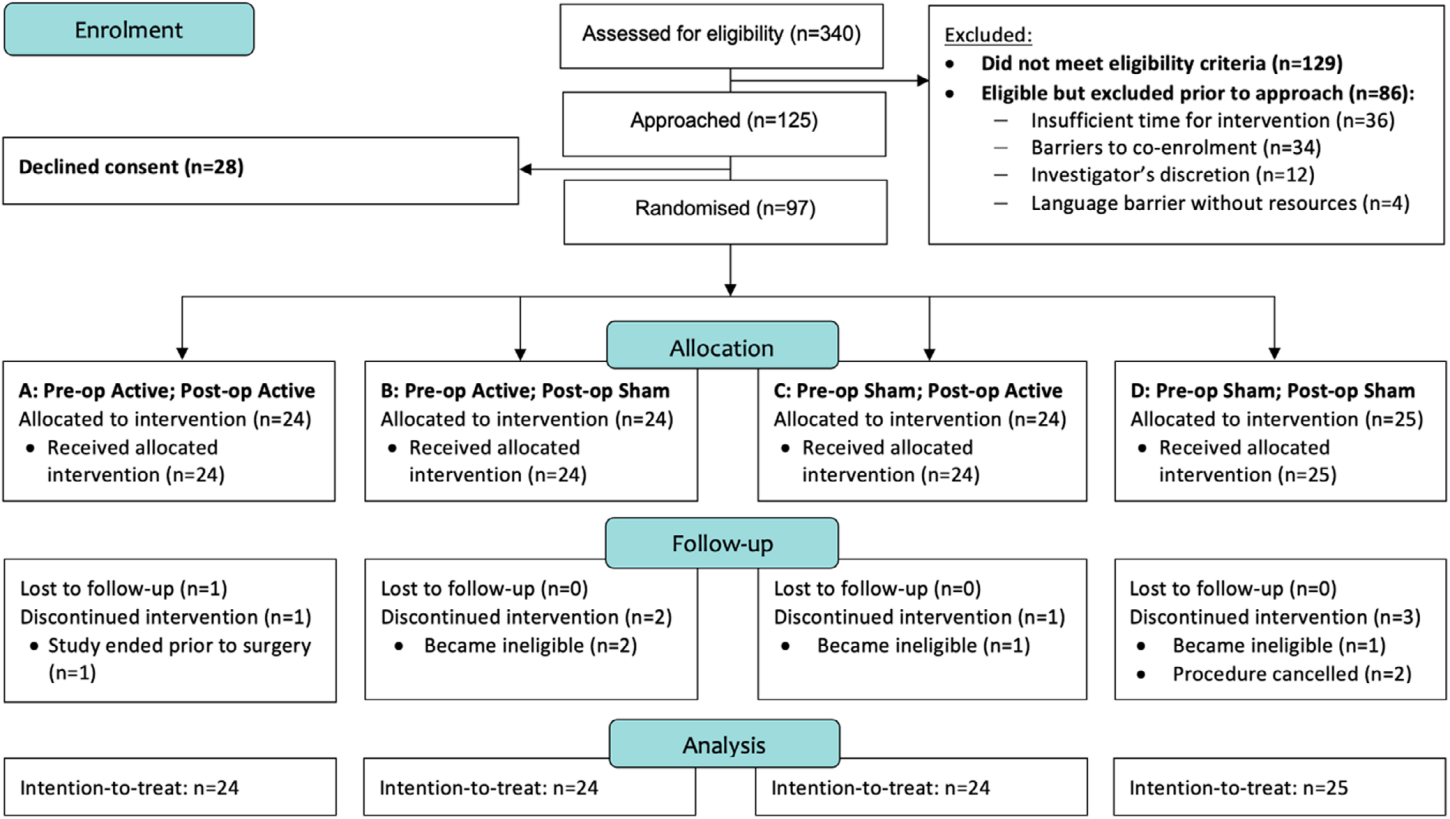
CONSORT diagram for recruitment to the feasibility trial.

A total of 340 patients were considered for participation. Of these, 211 (*n* = 211/340; 62.1%) were eligible, 125 (*n* = 125/211; 59.2%) were approached, 97 (*n* = 97/125; 77.6%) were randomized, and one (*n* = 1/97; 1.0%) was lost to follow‐up (Figure 2). A small majority were men (*n* = 51/97; 52.6%) and the mean age was 65.7 (SD 9.2) years. Most procedures started laparoscopic (*n* = 86/97; 88.7%) or robotic (8/97; 8.2%). A full outline of participant characteristics is provided in Table 2.

**TABLE 2:** Summary of participant and operative characteristics.

	Group 1: Stim/stim *n* = 24	Group 2: Stim/sham *n* = 24	Group 3: Sham/stim *n* = 24	Group 4: Sham/sham *n* = 25	Total *n* = 97
Sex					
Male	16 (66.7%)	5 (20.8%)	18 (75.0%)	12 (48.0%)	51 (52.6%)
Female	8 (33.3%)	19 (79.2%)	6 (25.0%)	13 (52.0%)	46 (47.4%)
Age (years)	65.5 (7.1)	64.3 (12.1)	67.3 (7.2)	65.6 (9.7)	65.7 (9.2)
BMI (kg/m^2^)	30.0 (7.7)	27.9 (5.2)	28.9 (5.1)	28.6 (7.2)	28.9 (6.3)
Current smoker	2 (8.3%)	2 (8.3%)	2 (8.3%)	1 (4.0%)	7 (7.2%)
Prior abdominal surgery	6 (25.0%)	9 (37.5%)	11 (45.8%)	11 (44.0%)	37 (38.1%)
Ischaemic heart disease	1 (4.2%)	1 (4.2%)	0 (0.0%)	0 (0.0%)	2 (2.1%)
Diabetes mellitus	3 (12.5%)	1 (4.2%)	3 (12.5%)	1 (4.0%)	8 (8.2%)
Chronic kidney disease	1 (4.2%)	0 (0.0%)	1 (4.2%)	0 (0.0%)	2 (2.1%)
COPD	1 (4.2%)	1 (4.2%)	4 (16.7%)	1 (4.0%)	7 (7.2%)
Peripheral vascular disease	0 (0.0%)	0 (0.0%)	1 (4.2%)	1 (4.0%)	2 (2.1%)
Regular opioid use	0 (0.0%)	1 (4.2%)	2 (8.3%)	0 (0.0%)	3 (3.1%)
Baseline Hb (g/L)	142.6 (17.2)	133.4 (18.7)	137.5 (14.9)	131.9 (16.0)	136.3 (17.0)
Baseline albumin (g/L)	40.2 (8.6)	38.4 (2.8)	37.6 (3.5)	38.5 (3.3)	38.6 (5.0)
Baseline eGFR^a^	77.5 (13.6)	80.7 (11.0)	83.3 (11.5)	81.4 (12.4)	80.8 (12.2)
ASA					
1	4 (16.7%)	4 (16.7%)	4 (16.7%)	5 (20.0%)	17 (17.5%)
2	10 (41.7%)	16 (66.7%)	13 (54.2%)	17 (68.0%)	56 (57.7%)
3	8 (33.3%)	4 (16.7%)	6 (25.0%)	3 (12.0%)	21 (21.6%)
4	1 (4.2%)	0 (0.0%)	0 (0.0%)	0 (0.0%)	1 (1.0%)
Unavailable	1 (4.2%)	0 (0.0%)	1 (4.2%)	0 (0.0%)	2 (2.1%)
Operative approach					
Laparoscopic	20 (83.3%)	20 (83.3%)	23 (95.8%)	23 (92.0%)	86 (88.7%)
Robotic	2 (8.3%)	3 (12.5%)	1 (4.2%)	2 (8.0%)	8 (8.2%)
Open	1 (4.2%)	1 (4.2%)	0 (0.0%)	0 (0.0%)	2 (2.1%)
Unknown	1 (4.2%)	0 (0.0%)	0 (0.0%)	0 (0.0%)	1 (1.0%)
Conversion to open^b^					
Yes	3 (13.0%)	3 (13.0%)	0 (0.0%)	5 (20.0%)	11 (11.6%)
No	19 (82.6%)	20 (87.0%)	24 (100.0%)	20 (80.0%)	83 (87.4%)
Unavailable	1 (4.3%)	0 (0.0%)	0 (0.0%)	0 (0.0%)	1 (1.1%)
Procedure					
Ileocaecal resection	0 (0.0%)	1 (4.2%)	0 (0.0%)	1 (4.0%)	2 (2.1%)
Right hemicolectomy	6 (25.0%)	8 (33.3%)	9 (37.5%)	8 (32.0%)	31 (32.0%)
Ext right hemicolectomy	4 (16.7%)	1 (4.2%)	3 (12.5%)	3 (12.0%)	11 (11.3%)
Transverse colectomy	0 (0.0%)	0 (0.0%)	0 (0.0%)	0 (0.0%)	0 (0.0%)
Left hemicolectomy	4 (16.7%)	3 (12.5%)	1 (4.2%)	2 (8.0%)	10 (10.3%)
Sigmoid colectomy	3 (12.5%)	1 (4.2%)	0 (0.0%)	5 (20.0%)	9 (9.3%)
Anterior resection	6 (25.0%)	10 (41.7%)	10 (41.7%)	6 (24.0%)	32 (33.0%)
Other	0 (0.0%)	0 (0.0%)	1 (4.2%)^c^	0 (0.0%)	1 (1.0%)
Unavailable	1 (4.2%) Ω	0 (0.0%)	0 (0.0%)	0 (0.0%)	1 (1.0%)
Unplanned stoma	0 (0.0%)	2 (8.3%)	3 (12.5%)	3 (12.0%)	8 (8.2%)
Duration of surgery (min)	203 (64)	166 (55)	195 (51)	191 (73)	190 (62)

Abbreviations: ASA, American Society of Anesthesiologists; BMI, body mass index; COPD, chronic obstructive pulmonary disease; eGFR, estimated glomerular filtration rate; Hb, haemoglobin.

^a^ Units are mL/min/1.73 m^2^.

^b^ Values exclude *n* = 2 participants (Group 1, *n* = 1; Group 2, *n* = 1) whose surgery started open.

^c^ Abandoned procedure without resection; Ω, surgery postponed beyond closure date of study.

### Feasibility of recruitment

Ninety‐seven out of the 125 patients approached about the study consented to take part. The remaining 28 declined, most commonly due to excess burden (*n* = 14/28; 50.0%) and the feeling of being too unwell (*n* = 3/28; 10.7%) (Table S1). Across both hospitals, the median number of participants randomized per month was four (interquartile range [IQR] 2–6; range 0–8). Overall, ‘Modify’ and ‘Go’ outcomes predominated across the recruitment period at each site. A full outline of hospital‐specific recruitment is provided in Figure S1.

### Feasibility of self‐administration

Across all groups, the median compliance to treatment was 19 out of 20 self‐administered stimulations (IQR 17–20), with a median preoperative compliance of 10 out of 10 (IQR 10–10) and postoperative compliance of 10 out of 10 (IQR 8–10). Compliance across treatment groups was similar, with median compliances of 20 (IQR 17–20), 17 (13, 14, 15, 16, 17, 18, 19, 20), 19 (18, 19, 20) and 20 (19, 20) for Group 1 (+, +), Group 2 (+, –), Group 3 (−, +) and Group 4 (−, –), respectively (Figure S2).

### Feasibility of treatment blinding

A total of 96 out of 97 participants completed the blinding assessment, with the remaining participant lost to follow‐up. Participant blinding in Group 1 (+, +) and Group 4 (−, –) demonstrated ‘Modify’ feasibility outcomes with Bang blinding indices of 0.40 and −0.27, respectively. This contrasted with Group 2 (+, –), and Group 3 (−, +), which both demonstrated ‘Stop’ outcomes with indices of 0.67 and 0.84, implying high rates of unblinding (Table S2).

### Return of intestinal function

**FIGURE 3:**
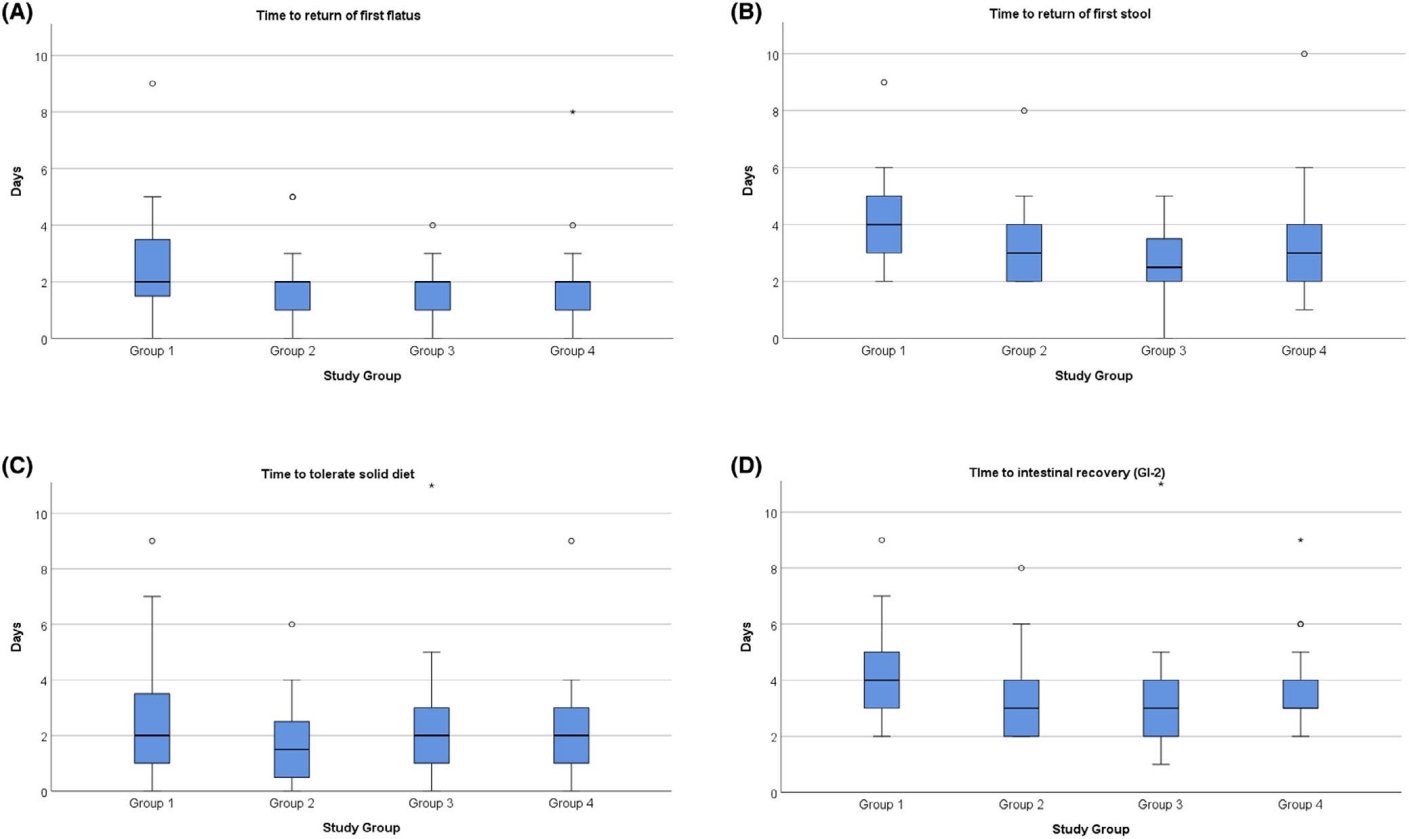
Return of intestinal function: (A) time to return of first flatus; (B) time to return of first stool; (C) time to tolerate solid diet; (D) time to intestinal recovery (GI‐2, a composite measure of time to first stool and oral diet). Group 1: Preoperative stimulation/postoperative stimulation. Group 2: Preoperative stimulation/postoperative sham. Group 3: Preoperative sham/postoperative stimulation. Group 4: Preoperative sham/postoperative sham.

The time taken for the return of intestinal function was similar across groups. The median number of days to first stool was 4 (IQR 3, 4, 5), 3 (IQR 2, 3, 4), 2.5 (IQR 2–3.5) and 3 (IQR 2, 3, 4) and the median days to tolerate solid diet was 2 (IQR 1–3.5), 1.5 (IQR 0.5–2.5), 2 (IQR 1, 2, 3) and 2 (IQR 1, 2, 3) for Group 1 (+, +), Group 2 (+, –), Group 3 (−, +) and Group 4 (−, –), respectively. Eighteen (*n* = 18/97; 18.6%) participants required a nasogastric tube after surgery, including eight (*n* = 8/24; 33.3%), four (*n* = 4/24; 16.7%), four (*n* = 4/24; 16.7%) and two (*n* = 2/25; 8.0%), respectively. The median hospital stay was 6 (IQR 4.5–10), 5 (IQR 3.75–8), 6 (IQR 3.75–8) and 4 (IQR 3, 4, 5, 6) days, respectively. A full outline of clinical outcomes is shown in Figure 3.

### Safety and adverse events

Postoperative complications occurred in 46 of 97 (47.4%) participants, including 10 in Group 1 (+, +) (*n* = 10/24; 41.7%), 12 in Group 2 (+, −) (*n* = 12/24; 50.0%), 12 in Group 3 (−, +) (*n* = 12/24; 50.0%) and 12 in Group 4 (−, –) (*n* = 12/25; 48.0%). The most common were surgical site infection (*n* = 18/97; 18.6%) and pneumonia (*n* = 7/97; 7.2%). Device‐related adverse events were infrequent, with neck strain (*n* = 3/97; 3.1%) and stimulation site pain (*n* = 3/97; 3.1%) being the most common. A full description of safety data is shown in Table 3.

**TABLE 3:** Summary of device‐related adverse events and postoperative complications.

	Group 1: Stim/stim (*n* = 24)	Group 2: Stim/sham (*n* = 24)	Group 3: Sham/stim (*n* = 24)	Group 4: Sham/sham (*n* = 25)	Total (*n* = 97)
Device‐related adverse events					
Headache	0 (0.0%)	1 (4.2%)	0 (0.0%)	1 (4.0%)	2 (2.1%)
Stimulation site pain	0 (0.0%)	1 (4.2%)	2 (8.3%)	0 (0.0%)	3 (3.1%)
Tooth pain	0 (0.0%	0 (0.0%)	1 (4.2%)	0 (0.0%)	1 (1.0%)
Neck strain	0 (0.0%)	1 (4.2%)	1 (4.2%)	1 (4.0%)	3 (3.1%)
Hoarseness/change in voice	0 (0.0%)	0 (0.0%)	0 (0.0%)	0 (0.0%)	0 (0.0%)
Dry mouth/change in taste	0 (0.0%)	1 (4.2%)	1 (4.2%)	0 (0.0%)	2 (2.1%)
Skin irritation	0 (0.0%)	0 (0.0%)	0 (0.0%)	1 (4.0%)	1 (1.0%)
Postoperative complications					
Acute coronary syndrome	0 (0.0%)	0 (0.0%)	0 (0.0%)	0 (0.0%)	0 (0.0%)
Acute kidney injury	2 (8.3%)	1 (4.2%)	1 (4.2%)	0 (0.0%)	4 (4.1%)
Anastomotic leak	1 (4.2%)	1 (4.2%)	1 (4.2%)	1 (4.0%)	4 (4.1%)
Cardiac arrythmia	1 (4.2%)	3 (12.5)	1 (4.2%)	0 (0.0%)	5 (5.2%)
Cerebrovascular accident	0 (0.0%)	0 (0.0%)	0 (0.0%)	0 (0.0%)	0 (0.0%)
Postoperative collection	2 (8.3%)	0 (0.0%)	0 (0.0%)	0 (0.0%)	2 (2.1%)
Pneumonia	5 (20.8%)	1 (4.2%)	1 (4.2%)	0 (0.0%)	7 (7.2%)
Surgical site infection	3 (12.5%)	5 (20.8%)	7 (29.2%)	3 (12.0%)	18 (18.6%)
Urinary tract infection	0 (0.0%)	1 (4.2%)	2 (8.3%)	1 (4.0%)	4 (4.1%)
Venous access infection	0 (0.0%)	0 (0.0%)	0 (0.0%)	0 (0.0%)	0 (0.0%)
Venous thrombo‐embolism	0 (0.0%)	0 (0.0%)	0 (0.0%)	1 (4.0%)	1 (1.0%)
Other					
Electrolyte disturbance^a^	1 (4.2%)	0 (0.0%)	1 (4.2%)	1 (4.0%)	3 (3.1%)
Intestinal obstruction	0 (0.0%)	0 (0.0%)	0 (0.0%)	1 (4.0%)	1 (1.0%)
Loose stools	0 (0.0%)	1 (4.2%)	1 (4.2%)	4 (16.0%)	6 (6.2%)
Omental infarct	1 (4.2%)	0 (0.0%)	0 (0.0%)	0 (0.0%)	1 (1.0%)
Pleural effusion	1 (4.2%)	0 (0.0%)	0 (0.0%)	0 (0.0%)	1 (1.0%)
Rectal bleeding	2 (8.3%)	2 (8.3%)	0 (0.0%)	1 (4.0%)	5 (5.2%)
Splenic infarct	1 (4.2%)	0 (0.0%)	0 (0.0%)	0 (0.0%)	1 (1.0%)
Vaso‐vagal syncope	0 (0.0%)	0 (0.0%)	0 (0.0%)	1 (4.0%)	1 (1.0%)
Surgical site bleeding	0 (0.0%)	2 (8.3%)^b^	0 (0.0%)	0 (0.0%)	2 (2.1%)
Wound dehiscence	0 (0.0%)	1 (4.2%)	0 (0.0%)	1 (4.0%)	2 (2.1%)
Unplanned critical care	2 (8.3%)	0 (0.0%)	0 (0.0%)	1 (4.0%)	3 (3.1%)
Unplanned readmission	0 (0.0%)	3 (12.5%)	2 (8.3%)	0 (0.0%)	5 (5.2%)
Clavien–Dindo classification					
Grade 1–2	9 (37.5%)	12 (50.0%)	12 (50.0%)	11 (44.0%)	44 (45.4%)
Grade 3–4	3 (12.5%)	0 (0.0%)	1 (4.2%)	2 (8.0%)	6 (6.2%)
Grade 5	0 (0.0%)	0 (0.0%)	0 (0.0%)	0 (0.0%)	0 (0.0%)

^a^ Includes hypokalaemia (*n* = 2) and hypophosphataemia (*n* = 1).

^b^ Includes wound site (*n* = 1) and peristomal bleeding (*n* = 1).

### Participant interviews

Interviews with 19 patients and 10 healthcare professionals were completed (Tables S3 and S4), generating four major themes of discussion per group (Table S5).

Good compliance to nVNS was a product of patients' motivation to contribute actively to their recovery after surgery. Feelings of being overwhelmed with surgery and their diagnosis was a barrier for a small number who chose not to take part.

‘I was keen on anything that I could do to get back going again’ (Patient 16, 61F, Group 2)

‘It [was] too much with what I was going through…’ (Patient 17, 64F, non‐participant)

Some participants experienced an initial learning curve whilst using the device. A key challenge was identifying the carotid pulse and using it as a landmark for the vagus nerve. This was compounded by logistical challenges in hospital, such as tubes and equipment. This did not impact on compliance, but showed the importance of good training to ensure treatment fidelity.

‘The biggest problem was finding my pulse…’ (Patient 8, 74F, Group 1)

‘It's not so easy to find [the pulse] at times. And there were times when I had an oxygen thing round me as well, which was getting in the way…’ (Patient 3, 78M, Group 4)

‘I think once you've done it once or twice, it just came naturally’(Patient 25, 50M, Group 4)

‘…face‐to‐face [training] was a big help because I knew what the device was and where it was going’ (Patient 15, 62M, Group 2)

Participants exposed to both types of devices (Groups 2 and 3) were commonly unblinded, mainly due to noticeable differences in their experience using the devices. In keeping, participants exposed to only one type of device (Groups 1 and 4) remained blinded appropriately.

‘The first device that I had before the [operation] seemed to be a lot more powerful than the second one’ (Patient 15, 62M, Group 2)

‘I could not tell the difference between the two devices. It might have been the same device for all I knew’(Patient 1, 66M, Group 1)

Amongst healthcare professionals, there was a strong aspiration to tackle the problem of ileus, as well as recognition of its profound impact on patients.

‘I think ileus is the last big thing that we don't understand and don't have a great strategy to offset and anticipate in our patients’ (Surgeon 3, Female, Non‐study site)

‘I've had patients in the past say they'd rather die than have another ileus! So, you know… it is really debilitating for them’ (Specialist Nurse 2, Female, Non‐study site)

A key barrier to implementing nVNS in practice was anxiety about its burden on staff workload. This was considered minimal owing to patients' role in administering the device independently.

‘You're not asking the surgeon to do anything… it's a patient‐driven intervention, so you might find implementation is better…’ (Surgeon 1, Male, Non‐study site)

‘They took, I suppose, a certain degree of ownership of it… I think it's—it's a real lesson in experience of patients…’ (Research Nurse 1, Male, Feasibility site)

## DISCUSSION

Non‐invasive vagus nerve stimulation has emerged as a new candidate treatment to reduce ileus after colorectal surgery. Preclinical work has demonstrated a clear anti‐inflammatory mechanism leading to faster gut recovery in rodents, with early clinical studies also confirming an acceptable safety profile after surgery. This feasibility study shows that patients are readily prepared to enrol in a trial of nVNS and are highly compliant to self‐administration. Preliminary assessments did not show a signal of efficacy across measures of intestinal function, although the study was not designed to statistically compare these. Interviews with patients and health professionals informed key refinements necessary for a randomized trial of nVNS in the future.

This study adds to a small body of evidence exploring the efficacy of nVNS for reducing ileus after surgery. Hong and colleagues showed that stimulating the auricular branch of the vagus nerve once for 10 min during open surgery (frequency 25 Hz; current 10 mA) led to significant changes in gastric muscle activity. This was seen as a reduction in frequency and an increase in amplitude of action potentials, supporting the hypothesis that vagal stimulation improves gastric propulsion [11]. In a randomized study of 134 patients undergoing laparoscopic surgery, Ru and colleagues demonstrated a reduction in ileus (6% vs. 20%; *P* = 0.022) using auricular vagus nerve stimulation. This was performed once for 20 min prior to anaesthesia (25 Hz; 10 mA) [23]. In our earlier proof‐of‐concept work, we provided an early experience of cervical nVNS after surgery using the gammaCore device (25 Hz; peak 60 mA), demonstrating a trend towards reduced time to first stool (2.35 ± 1.32 vs. 1.65 ± 0.88 days) following twice‐daily stimulation [12]. Importantly, none of these studies were adequately powered to definitively evaluate the effect of nVNS on intestinal function. It is probable that the disparity between the results of the present and previous work is due to small sample sizes across studies, which justifies a definitive, statistically powered study to determine whether nVNS is efficacious for improving ileus after surgery or not.

The decision to use the present stimulation parameters (25 Hz; peak 60 mA) was guided by earlier experimental evidence using the gammaCore device. Studies of healthy volunteers previously demonstrated significant reductions in markers of systemic inflammation (interleukin 1β and tumour necrosis factor α) as well as elevated measures of cardiac vagal tone 24 h after stimulation. These data confirm that the device has an intended physiological effect in humans when the vagus nerve is stimulated non‐invasively [24, 25]. No evidence existed to inform the overall duration of treatment aside from our proof‐of‐concept work. A schedule of 5 days before and after surgery was selected to encompass the typical time taken for return of bowel function, along with a nominal preoperative time period for patients to build familiarity with the device.

This feasibility assessment provided key insights for translating nVNS to future trials and clinical practice. It showed that recruiting a complex population undergoing major oncological surgery, along with the attendant physical and emotional burdens, was readily feasible [26]. It showed that compliance to a self‐administered treatment in the perioperative period was achievable, driven by participants' strive for autonomy in their care [27]. The findings also highlighted opportunities to maximize feasibility, most notably participants' ability to identify their carotid pulse. Interviews highlighted the importance of face‐to‐face training with practical demonstrations to improve confidence. Device blinding was also challenging, with unblinding occurring in treatment groups where participants were exposed to both types of device [28].

A strength of this work is the inclusion of interviews with patients and health professionals, which provided valuable data to contextualize the findings. Alongside the feasibility trial, this enabled an assessment of barriers and opportunities to translate nVNS from preclinical to future trials. Limitations are also recognized. Most importantly, the study represents unpowered feasibility work designed to inform definitive research in the future. As such, it is not able to confirm or refute arguments for clinical efficacy. Secondly, owing to the observed learning curve associated with self‐administering the device, optimization of training processes is the next interval step to be undertaken in collaboration with public representatives. Next, it is acknowledged that per‐participant compliance to fast‐track recovery protocols is a key consideration for future studies which was not considered in the present work. Lastly, it is recognized that the selection of clinical outcomes for a definitive study must be relevant to patients and health professionals alike. This will be guided by a recently agreed core outcome set for ileus, developed through international consensus [29].

In conclusion, a definitive evaluation of nVNS to reduce ileus after colorectal surgery is feasible once the approach to training is optimized. There is strong enthusiasm from health professionals to tackle the problem of ileus, as well as enthusiasm amongst patients for interventions which engage them actively in their recovery. Current evidence around nVNS should progress from its focus on early phase assessment towards randomized evaluations of efficacy and, in turn, towards evaluations of clinical effectiveness. Drawing on all data available, it is expected that this will comprise a parallel‐group design, assessing previously agreed core outcomes [29]. Incorporating the intervention within a master trial protocol, such as a platform trial for ileus, could provide an efficient and favourable approach. A patient‐reported outcome measure is currently in development and is a key candidate for a primary outcome assessment [30].

## AUTHOR CONTRIBUTIONS

**Stephen J. Chapman:** Conceptualization; methodology; data curation; investigation; formal analysis; funding acquisition; project administration; resources; writing – original draft. **Mikolaj Kowal:** Data curation; investigation; writing – review and editing. **Jack A. Helliwell:** Data curation; investigation; writing – review and editing. **Sonia Lockwood:** Data curation; investigation; writing – review and editing. **Maureen Naylor:** Methodology; writing – review and editing; investigation. **Julie Croft:** Methodology; resources; writing – review and editing. **Katherine Farley:** Methodology; formal analysis; supervision; writing – review and editing. **Deborah D. Stocken:** Methodology; formal analysis; supervision; writing – review and editing. **David G. Jayne:** Conceptualization; methodology; supervision; funding acquisition; writing – review and editing.

## FUNDING INFORMATION

The feasibility work was funded by a National Institute for Health and Care Research (NIHR) Doctoral Research Fellowship (S. Chapman; DRF‐2018‐11‐049). Study devices were provided through the electroCore Inc. Investigator Initiated Trial Programme. The study was supported by the NIHR Surgical MedTech Co‐operative.

## CONFLICT OF INTEREST STATEMENT

No competing interests are declared. The views expressed in this publication are those of the authors and not necessarily those of the NHS, the National Institute for Health and Care Research, Health Education England or the Department of Health.

## ETHICS STATEMENT

Research ethics approval for the feasibility trial was confirmed by the North East Tyne and Wear South NHS Research Ethics Committee (19/NE/0217) on 2 July 2019. Written informed consent to participate in each of the feasibility trial and qualitative interviews was obtained from all participants.

## CLINICAL TRIAL REGISTRATION NUMBER

The feasibility trial was prospectively registered on the ISRCTN register (ISRCTN62033341) on 11 October 2019 (http://www.isrctn.com/ISRCTN62033341).

## Supporting information


Data S1.
Table S1.
Table S2.
Table S3.
Table S4.
Table S5.
Figure S1.
Figure S2.

## Data Availability

Data sharing requests will be considered by the study management group on written request to the corresponding author. Fully anonymized participant data or other prespecified data will be available subject to a written proposal and a signed data‐sharing agreement.
