# Supplementary material for: Non‐invasive vagus nerve stimulation to reduce ileus after colorectal surgery: randomized feasibility trial and efficacy assessment (IDEAL Stage 2B)
Source: Colorectal Dis. 2024 Oct 12;26(12):2101–11. doi: 10.1111/codi.17194 (PMC11649866; doi:10.1111/codi.17194)
Supplement: Supplementary file 1 — Data S1. Table S1. Table S2. Table S3. Table S4. Table S5. Figure S1. Figure S2. [file CODI-26-2101-s001.docx]

**Appendix: Non-invasive vagus nerve stimulation to reduce ileus after intestinal surgery: feasibility and efficacy assessment**

[**SUPPLEMENTARY & EXPANDED METHODS** 2](#_Toc144740134)

[Ethics & Governance 2](#_Toc144740135)

[Variations to protocol 2](#_Toc144740136)

[Setting and Participants 3](#_Toc144740137)

[*Study Sites* 3](#_Toc144740138)

[*Eligibility Criteria* 3](#_Toc144740139)

[*Recruitment* 4](#_Toc144740140)

[Interventions 4](#_Toc144740141)

[*Study site processes* 4](#_Toc144740142)

[*Participant Training & Administration* 4](#_Toc144740143)

[Feasibility Study 5](#_Toc144740144)

[*Randomisation & Blinding* 5](#_Toc144740145)

[*Study Outcomes* 5](#_Toc144740146)

[*Sample Size & Data Analysis* 6](#_Toc144740147)

[Qualitative Interviews 7](#_Toc144740148)

[*Research team and reflexivity* 7](#_Toc144740149)

[*Theoretical framework* 7](#_Toc144740150)

[*Data collection* 8](#_Toc144740151)

[*Qualitative analysis* 8](#_Toc144740152)

**SUPPLEMENTARY TABLES AND FIGURES** 10

[Table S1 – Summary of reasons for declined consent from feasibility trial 1](#_Toc144740158)1

[Table S2 – Assessment of blinding performance (Modified Bang Blinding Index) 1](#_Toc144740159)2

[Table S3 – Characteristics of patients taking part in an interview 1](#_Toc144740161)3

[Table S4 – Characteristics of health professionals taking part in an interview 1](#_Toc144740162)4

[Table S5 – Summary of patient and health professional themes from interviews 1](#_Toc144740158)5

[Figure S1 – Cumulative participant recruitment charts for participating hospitals 1](#_Toc144740159)6

[Figure S2 – Self-administered nVNS compliance over time by group 1](#_Toc144740161)7

# Ethics & Governance

The trial was undertaken according to the Declaration of Helsinki and the International Conference on Harmonisation Good Clinical Practice Guidelines. Approval by the NHS Health Research Authority (HRA) and the Tyne & Wear South Research Ethics Committee (REC) was confirmed on 2^nd^ July 2019 (19/NE/0217). The study was registered on the ISRCTN registry on 11^th^ October 2019 prior to the start of enrolment (ISRCTN62033341). The study was funded by the National Institute of Health and Care Research (NIHR) and devices were provided through the electroCore Inc. Investigator Initiated Trial Programme. Neither had a role in the study design, data analysis, or interpretation of results.

# Variations to protocol

In 2020, coronavirus disease 2019 (COVID-19) caused by the severe acute respiratory syndrome coronavirus 2 was declared as a global pandemic by the World Health Organisation. The study was temporarily suspended between 18^th^ March 2020 and 1^st^ July 2020, in line with national guidance on the prioritisation of COVID-19 research, after which participating sites were invited to re-open recruitment in line with local operating procedures and risk assessments. Owing to subsequent social distancing requirements, a substantial amendment to the prospective protocol was approved on 23^rd^ June 2020 to enable all participant recruitment and intervention training activities to be performed by telephone. These processes were designed in close collaboration with public and patient representatives. The amended two-step consent process comprised an initial process of audio-recorded consent via telephone followed by written confirmation upon admission to hospital. The amended intervention training session comprised a telephone conversation using a dedicated step-by-step visual instruction guide as a training aid.

# Setting and Participants

## Study Sites

The trial was undertaken at St. James’s University Hospital (SJUH) in Leeds and Bradford Royal Infirmary (BRI) in Bradford. SJUH is a tertiary-care centre serving a local population of 812,000 (16% Aged 65 and over; 18.9% Black and Minority Ethnic groups) as well as a wider population of approximately 5 million across the West Yorkshire region (1). BRI is a secondary-care centre serving a local population of 546,000 (15.2% Aged 65 and over; 36.1% Black and Minority Ethnic groups) (2). Both provide planned minimally-invasive colorectal surgery within programmes of enhanced recovery.

## Eligibility Criteria

Patients were eligible to take part if they were aged 18 or over, able to provide written informed consent, and were due to undergo elective minimally invasive (laparoscopic or robotic) colorectal resection with no routine plans for a diverting stoma. Intraoperative decisions to convert to open surgery or to form an unplanned stoma did not lead to exclusion provided that these decisions were made after randomisation. Patients were excluded if they satisfied any of the following criteria: 1) severe cardiac disease (myocardial infarction within 12 months; congestive heart failure with New York Heart Association Scale > 2, second- or third-degree atrioventricular block, atrial fibrillation/flutter or previous ventricular tachycardia or fibrillation); 2) seizures or recurrent syncope in the last 5 years; 3) previous transient ischaemic attack or cerebrovascular accident; 4) previous vagotomy; 5) inflammatory bowel disease, 6) neuroendocrine tumour, 7) existing intestinal stoma, 8) implanted electrical device; 9) structural abnormality of the neck precluding administration of the device; 10) belonging to a vulnerable group 11) patients who were pregnant or nVNS, factors which may impact on its mechanism (such as previous vagotomy), and factors which may significantly impede on its administration (such as structural abnormality of the neck anatomy). Co-enrolment to other studies was permitted unless the intervention aimed to modify the trajectory of recovery or its action was considered to affect bowel motility.

## Recruitment

Potential participants were identified from multi-disciplinary team meetings and screened for eligibility according to pre-defined eligibility criteria. According to the original study protocol, they were approached in person and informed consent was confirmed following a final assessment of eligibility. Following the COVID-19 amendment, participants could also provide consent via telephone, which was later confirmed in writing at the time of hospital admission. Withdrawal from the study was permitted at any time if requested by participants.

# Interventions

## Study site processes

Study devices were introduced to sites according to local approval processes. Local investigators attended a study initiation visit prior to the start of recruitment. This involved a summary presentation of the study protocol, a demonstration of the device, and an introduction to the standard operating procedure for participant training. Devices were stored at local sites according to the manufacturer’s standard instructions.

## Participant Training & Administration

After enrolling in the trial, participants attended a face-to-face or telephone training session. This included a practical demonstration on how to locate the surface landmark of the cervical vagus nerve using the carotid pulse and how to activate and adjust the stimulation amplitude. Participants were invited to self-administer the device under supervision using a demonstration (inactive) device until they felt confident. A “Quick Guide” resource comprising of step-by-step written and visual instructions was provided to take home.

Participants self-administered the device twice-daily for five consecutive days both before and after surgery. The timing of administration was not pre-specified but participants were instructed to perform one administration in the morning and another approximately 12 hours later. Each administration comprised a 2-minute cycle of continuous stimulation which was performed sequentially on each side of the neck. The device shut down automatically after two minutes. Participants were instructed to apply conductive gel to the stimulation surfaces and to adjust the stimulation amplitude to the highest tolerated level.

# Feasibility Study

## Randomisation & Blinding

A computer-generated random allocation sequence was developed by an independent statistician at the Leeds Clinical Trials Unit. Randomisation was performed by minimisation with two stratification variables, including the type of surgery (right-sided or left-sided resection) and study site (SJUH or BRI). Local investigators randomised participants using an automated 24-hour online service and were also responsible for assigning patients to the randomised group. Participants were blinded to the treatment allocation through the use of an identical sham device which was identical in appearance, weight, audio feedback, and packaging. Local investigators and outcome assessors were not blinded to the allocation. Training activities and written materials were identical across all treatment groups. The pre-operative assigned device was replaced with a new post-operative device immediately after surgery. This took place routinely, irrespective of whether the same type of device was assigned before and after surgery to ensure standardisation of the study processes.

## Study Outcomes

The following outcomes were assessed to explore the feasibility of a definitive study:

- Proportion of eligible patients identified from screening logs per month
- Number of eligible patients recruited per month and reasons for approach failure
- Adequacy of participant blinding according to a modified Bang Index (3)
- Average compliance to the study treatment according to participant-reported diaries (expressed as a proportion of 20 stimulation cycles across 10 days)
- Rate of missing clinical endpoint data
- Rate of participant loss-to-follow up after enrolment in the study
- Incidence of postoperative complications occurring within 30 days of surgery

The following clinical outcomes were assessed to explore the promise of nVNS for reducing ileus after major intestinal surgery:

- Time (days) to first passage of flatus
- Time (days) to first passage of stool
- Time (days) to tolerate oral intake
- Time (days) to GI-2 (composite of oral intake and passage of stool) (4)
- Need for insertion of a nasogastric tube
- Total length of inpatient hospital stay (days)

## Sample Size & Data Analysis

A maximum sample size of 35 participants per study arm (total sample: 140) was considered appropriate to explore the feasibility outcomes and to assess the clinical promise if nVNS. This was guided by Teare and colleagues, who proposed that an external pilot study should include at least 35 participants in each arm and at least 70 in the study overall (5). There were no plans to stop the trial early and the end of the trial was considered to be when the planned sample was achieved or when 24 months of recruitment had elapsed. All feasibility and clinical outcomes were presented descriptively as rates (categorical) or means (continuous). As a feasibility study, no statistical comparisons across study arms were planned or undertaken. Blinding was assessed using a modified Bang Blinding Index. The original index ($BI)$ described by Bang provides an assessment of blinding for a two-arm RCT comprising, expressed as a value between -1 and 1 with 0 representing random guessing, as follows:

$BI= \frac{n_{c}-n_{i}}{n_{ci}}$ (E.2.1)

where $n_{c}$ represents correct guesses, $n_{i}$ represents incorrect guesses, and $n_{ci}$ represents the total number of responses received.

Since multiple arms existed in this study and it was possible for ‘incorrect’ guesses to be spread across any number of arms, the index was modified $({BI}_{m})$ as follows:

${BI}_{m}=\frac{n_{c}-\bar{n_{x}}}{{(n}_{c}+ \bar{n_{x}})}$ (E.2.2)

where $n_{c}$ represents correct guesses, $\bar{n_{x}}$ represents the average of incorrect guesses across all other arms, and $n_{c}+ \bar{n_{x}}$ was the respective modified total, thus normalising the equation to produce a comparable output to the original index.

# Qualitative Interviews

## Research team and reflexivity

All interviews were performed by a male investigator (SC). This investigator was a medical doctor with a background of surgical training. He held a basic postgraduate research training qualification and had experience of facilitating focus groups and interviews with health service users. He introduced himself to all participants as a researcher but gave no further detail about his medical background or reasons for doing the study unless specifically asked.

## Theoretical framework

The Theoretical Framework of Acceptability (TFA) was considered when undertaking this study. This describes a series of constructs that capture key dimensions of acceptability when developing, evaluating, and implementing healthcare interventions in practice. The constructs include: affective attitude, burden, ethicality, perceived effectiveness, intervention coherence, opportunity costs, and self-efficacy (6). The framework was used to develop semi-structured topic guides used during interviews with patients and healthcare professionals.

## Data collection

All participants took part in a single, semi-structured interview following additional confirmation of consent. Patient- and healthcare professional-specific topic guides were used to explore key issues but remained flexible and responsive to new ideas. For patient-participants, interviews took place after hospital discharge and for healthcare professional-participants they took place during the course of recruitment to the feasibility trial. A combination of telephone and face-to-face interviews were initially planned but these were converted to telephone in light of social distancing restrictions related to COVID-19. Interviews lasted between 17:03 and 53:28 minutes and were digitally recorded, pseudo-anonymised, and transcribed verbatim in preparation for analysis. Field notes were recorded by the investigator to add context to the data and to facilitate reflexive thinking.

## Qualitative analysis

A thematic framework analysis of interview transcripts was undertaken. Transcripts from patient and health professional participants were analysed separately owing to unique perspectives anticipated from each group. A single investigator reviewed all transcripts to build familiarity with the data and to develop initial coding frameworks. A second researcher independently reviewed two transcripts followed by a discussion of findings as a means of validation. The final frameworks were used to construct draft themes through a process of graphical mapping and cross-comparison. The themes were iteratively adapted and finalised with consideration to between-theme relationships. Data were summarised using graphical maps and quotations. No dedicated analysis software was used during the analysis process.

# Supplementary References

1. Leeds Observatory. Population Leeds. Available at: https://observatory.leeds.gov.uk/population/#/view-report/63aeddf1d7fc44b8b4dffcd868e84eac/___iaFirstFeature/G3 [Accessed 21st Oct 2022].
2. City of Bradford Metropolitan District Council. 2021 Census: Bradford District. Available at: https://ubd.bradford.gov.uk/about-us/2021-census/ [Accessed 21st Oct 2022].
3. Bang H. Ni L. Davis CE. Assessment of blinding in clinical trials. *Control Clin Trials*. 2004; 25:143-56.
4. van Bree SH. Bemelman WA. Hollmann MW. et al. . Identification of clinical outcome measures for recovery of gastrointestinal motility in postoperative ileus. *Ann Surg* 2014; 259: 708–714.
5. Teare MD. Dimario M. Shephard N. et al. Sample size requirements to estimate key design parameters from external pilot randomised controlled trials: a simulation study. *Trials* 2014;15:264.
6. Sekhon M. Cartwright M. Francis JJ. Acceptability of healthcare interventions: an overview of reviews and development of a theoretical framework. *BMJ Health Services Research* 2017; 17:88.
7. Chapman SJ. Helliwell JA. Naylor M. et al. Noninvasive vagus nerve stimulation to reduce ileus after major colorectal surgery: early development study. *Colorectal Dis* 2021;23:1225-1232.

**SUPPLEMENTARY TABLES & FIGURES**

# Table S1 – Summary of reasons for declined consent from feasibility trial

| Reason for declined consent | Total (n=28) |
| --- | --- |
| Patient felt too overburdened with clinical treatment | 14 (50.0%) |
| Patient was not contactable after approach to confirm participation | 3 (10.7%) |
| Patient considered him/herself too ill to take part | 3 (10.7%) |
| Patient was concerned about possible side-effect of the intervention | 2 (7.1%) |
| Patient was not interested in taking part in research | 1 (3.6%) |
| Participant did not feel confident with self-administering the device | 1 (3.6%) |
| Travel burden/time precluded enrolment | 1 (3.6%) |
| No reason given | 3 (10.7%) |

# Table S2 – Assessment of blinding performance (Modified Bang Blinding Index)

|  | Guess  _Group1_ | Guess  _Group2_ | Guess  _Group3_ | Guess  _Group4_ | Average of incorrect guesses ($\bar{n_{a}}$) | Total responses | ${BI}_{m}$ |
| --- | --- | --- | --- | --- | --- | --- | --- |
| Assigned_Group1_ | **10** | 7 | 3 | 3 | 4.33 (Groups: 2,3,4) | 23^*^ | 0.40 |
| Assigned_Group2_ | 1 | **15** | 5 | 3 | 3.00 (Groups: 1,3,4) | 24 | 0.67 |
| Assigned_Group3_ | 3 | 2 | **19** | 0 | 1.67 (Groups: 1,2,4) | 24 | 0.84 |
| Assigned_Group4_ | 7 | 8 | 6 | **4** | 7.00 (Groups: 1,2,3) | 25 | -0.27 |

*Group 1: Preoperative Stimulation/Postoperative Stimulation; Group 2: Preoperative Stimulation/Postoperative Sham; Group 3: Preoperative Sham/Postoperative Stimulation; Group 4: Preoperative Sham/Postoperative Sham;* ${BI}_{m}$ *: modified blinding*

*index; and* $\bar{n_{a}}$*: average incorrect guesses across all other groups, as per the Supplementary Methods.*

** A total of 23 out of 24 participants entered the analysis, with one participant lost to follow up*

# Table S3 – Characteristics of patients taking part in an interview

| Pseudonym | Intervention Group | Sex | Age | Type of Surgery |
| --- | --- | --- | --- | --- |
| Patient 1 | 1 (Stim/Stim) | Male | 66 | Right |
| Patient 2 | 4 (Sham/Sham) | Female | 64 | Right |
| Patient 3 | 4 (Sham/Sham) | Male | 78 | Right |
| Patient 4 | 2 (Stim/Sham) | Female | 61 | Left |
| Patient 5 | 3 (Sham/Stim) | Male | 76 | Right |
| Patient 6 | 2 (Stim/Sham) | Female | 81 | Right |
| Patient 7 | 2 (Stim/Sham) | Female | 65 | Right |
| Patient 8 | 1 (Stim/Stim) | Female | 74 | Right |
| Patient 9 | 3 (Sham/Stim) | Male | 60 | Left |
| Patient 10 | 1 (Stim/Stim) | Male | 55 | Left |
| Patient 11 | 3 (Sham/Stim) | Male | 77 | Left |
| Patient 12 | 4 (Sham/Sham) | Male | 50 | Right |
| Patient 13 | 3 (Sham/Stim) | Female | 69 | Right |
| Patient 14 | 4 (Sham/Sham) | Female | 83 | Left |
| Patient 15 | 2 (Stim/Sham) | Male | 62 | Left |
| Patient 16 | 2 (Stim/Sham) | Female | 61 | Right |
| Patient 17 | Non-participant* | Female | 64 | Left |
| Patient 18 | Non-participant* | Female | 67 | Left |
| Patient 19 | Non-participant* | Male | 70 | Left |

**Indicates interview participants who declined to take part in the feasibility trial (non-participant interviews);* *Group 1: preoperative stimulation; postoperative stimulation: Group 2: preoperative stimulation; postoperative sham; Group 3: preoperative sham; postoperative stimulation; Group 4: preoperative sham; postoperative sham.*

# Table S4 – Characteristics of health professionals taking part in an interview

| Pseudonym | Sex | Years since qualification | Type of Hospital | Study site |
| --- | --- | --- | --- | --- |
| Research Nurse1 | Male | >20 | Academic | Feasibility study site |
| Research Nurse2 | Female | >20 | District general | Feasibility study site |
| Specialist Nurse 1 | Female | 1-9 | Academic | Feasibility study site |
| Specialist Nurse 2 | Female | >20 | Academic | Non-study site |
| Specialist Nurse 3 | Female | >20 | Academic | Non-study site |
| Surgeon 1 | Male | 10-19 | District general | Non-study site |
| Surgeon 2 | Male | 10-19 | Academic | Non-study site |
| Surgeon 3 | Female | >20 | Academic | Non-study site |
| Surgeon 4 | Male | >20 | Academic | Non-study site |
| Surgeon 5 | Male | >20 | District general | Non-study site |

“Academic hospital” relates to an institution formally linked to a higher education institute or university. “Feasibility study site” relates to one of two hospitals which were involved in recruitment to the feasibility study and whose staff were closely familiar with the device. “Non-study site” refers to a hospital in the UK which was not involved in the feasibility study and whose staff were not closely familiar with the device.

| Patient themes | Health professional themes |
| --- | --- |
| Theme 1: Drivers and barriers to self-participation in recovery | Theme 1: Perspectives, knowledge, and experiences of ileus |
| Theme 2: Navigating the learning curve and mitigative strategies | Theme 2: The challenge of implementing vagus nerve stimulation |
| Theme 3: Developing confidence through familiarity and knowledge | Theme 3: Putting patient empowerment into practice during recovery |
| Theme 4: Investment and commitment to medical research | Theme 4: Overcoming barriers to recruitment |

**Table S5 – Summary of patient and health professional themes from interviews**


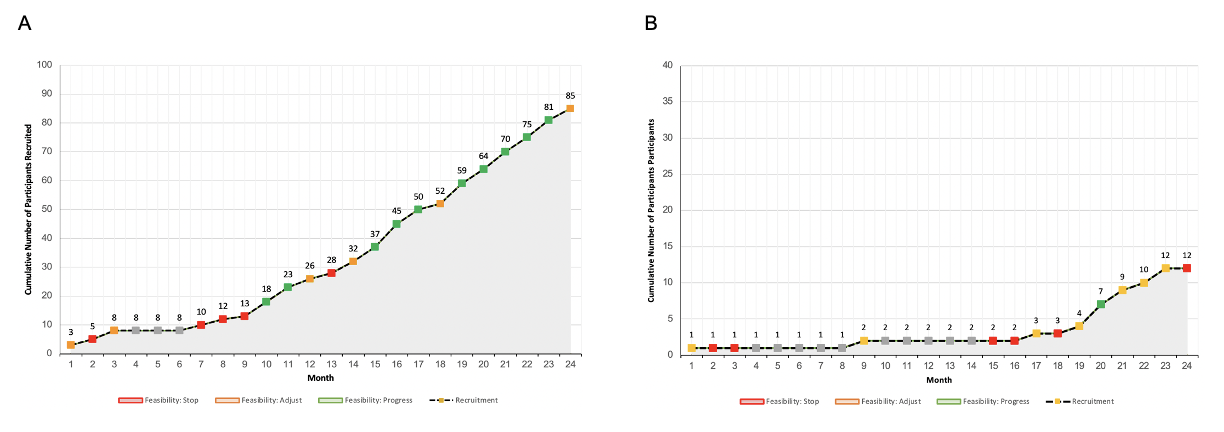
**Figure S1 – Cumulative participant recruitment charts for participating hospitals**

*A: Recruitment chart for St. James’s University Hospital, Leeds, UK; B: Recruitment chart for Bradford Royal Infirmary, Bradford, UK. Red, amber, and green squares indicate monthly recruitment outcomes according to the prospective progression criteria (Go, Modify, and Stop, respectively). Grey squares indicate months during which recruitment was temporarily suspended due to the COVID-19 pandemic*

# Figure S2 – Self-administered nVNS compliance over time by group


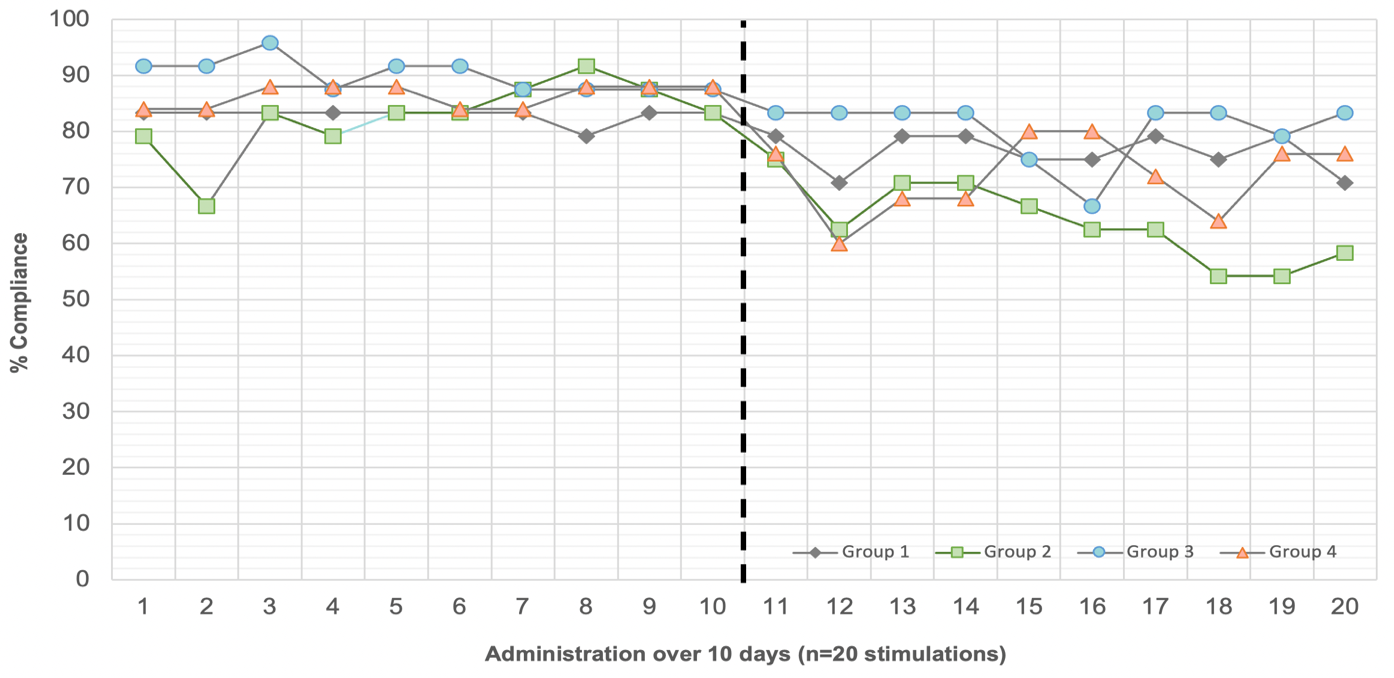


*Dashed line represents time of surgery and thus divides pre- and postoperative administration periods. Group 1: preoperative stimulation; postoperative stimulation: Group 2: preoperative stimulation; postoperative sham; Group 3: preoperative sham; postoperative stimulation; Group 4: preoperative sham; postoperative sham*
